# Supplementary material for: Hot spot prediction in protein-protein interactions by an ensemble system
Source: BMC Syst Biol. 2018 Dec 31;12(Suppl 9):132. doi: 10.1186/s12918-018-0665-8 (PMC6311905; doi:10.1186/s12918-018-0665-8)
Supplement: Supplementary file 2 — All relevant MATLAB code. A simple Matlab implement of our predictor. (ZIP 19,742 kb) [file 12918_2018_665_MOESM2_ESM.zip › Additional file 2/Read ASA/ASA website/dbMPIKT/4MYWA.html]

 NetSurfP - Protein Surface Accessibility and Secondary Structure Predictions

  

|  |  |  |
| --- | --- | --- |
|  |  | NetSurfP - Protein Surface Accessibility and Secondary Structure Predictions  Technical University of Denmark |

  


---

```
# For publication of results, please cite:
# A generic method for assignment of reliability scores applied to solvent accessibility predictions.
# Bent Petersen, Thomas Nordahl Petersen, Pernille Andersen, Morten Nielsen and Claus Lundegaard
# BMC Structural Biology 2009, 9:51 doi:10.1186/1472-6807-9-51
#
# Column 1: Class assignment - B for buried or E for Exposed - Threshold: 25% exposure, but not based on RSA
# Column 2: Amino acid
# Column 3: Sequence name
# Column 4: Amino acid number
# Column 5: Relative Surface Accessibility - RSA
# Column 6: Absolute Surface Accessibility
# Column 7: Z-fit score for RSA prediction
# Column 8: Probability for Alpha-Helix
# Column 9: Probability for Beta-strand
# Column 10: Probability for Coil
E A  4MYWA                  1    0.824  90.783  -0.576   0.003   0.003   0.994
E D  4MYWA                  2    0.704 101.374  -0.655   0.018   0.047   0.935
B L  4MYWA                  3    0.249  45.519  -0.317   0.056   0.142   0.802
E K  4MYWA                  4    0.581 119.532   1.002   0.066   0.296   0.638
B Y  4MYWA                  5    0.258  55.113   0.199   0.069   0.386   0.545
E A  4MYWA                  6    0.391  43.132   0.216   0.069   0.386   0.545
E L  4MYWA                  7    0.307  56.138  -0.929   0.069   0.386   0.545
B A  4MYWA                  8    0.287  31.649  -0.718   0.064   0.216   0.721
E D  4MYWA                  9    0.515  74.255  -0.221   0.052   0.084   0.864
E P  4MYWA                 10    0.412  58.434  -0.353   0.053   0.043   0.903
E S  4MYWA                 11    0.402  47.114  -0.290   0.064   0.216   0.721
B L  4MYWA                 12    0.107  19.555   0.305   0.079   0.592   0.329
E K  4MYWA                 13    0.373  76.747   0.403   0.023   0.655   0.322
E M  4MYWA                 14    0.316  63.272  -0.365   0.023   0.655   0.322
B A  4MYWA                 15    0.175  19.252  -1.042   0.021   0.451   0.528
E D  4MYWA                 16    0.381  54.888  -0.182   0.019   0.141   0.840
E P  4MYWA                 17    0.414  58.747  -1.064   0.018   0.019   0.964
E N  4MYWA                 18    0.640  93.637  -1.608   0.018   0.019   0.964
E R  4MYWA                 19    0.403  92.379   0.170   0.052   0.084   0.864
B F  4MYWA                 20    0.115  23.020  -1.160   0.056   0.142   0.802
E R  4MYWA                 21    0.445 101.859   0.177   0.064   0.216   0.721
B G  4MYWA                 22    0.321  25.302  -2.115   0.019   0.141   0.840
E K  4MYWA                 23    0.518 106.532   0.108   0.019   0.141   0.840
E N  4MYWA                 24    0.600  87.811  -0.937   0.004   0.085   0.910
B L  4MYWA                 25    0.108  19.830   0.543   0.018   0.047   0.935
E P  4MYWA                 26    0.545  77.335  -0.944   0.018   0.019   0.964
E V  4MYWA                 27    0.505  77.588  -0.442   0.018   0.019   0.964
B L  4MYWA                 28    0.125  22.796   0.412   0.018   0.019   0.964
E D  4MYWA                 29    0.592  85.293  -0.925   0.018   0.019   0.964
E Q  4MYWA                 30    0.533  95.122  -0.765   0.018   0.019   0.964
E L  4MYWA                 31    0.456  83.457  -0.998   0.018   0.019   0.964
E T  4MYWA                 32    0.528  73.303  -1.802   0.018   0.019   0.964
E D  4MYWA                 33    0.403  58.029  -1.061   0.005   0.015   0.979
B P  4MYWA                 34    0.240  34.013  -0.887   0.016   0.005   0.979
E P  4MYWA                 35    0.391  55.511  -0.972   0.058   0.017   0.925
E G  4MYWA                 36    0.593  46.645  -0.628   0.058   0.017   0.925
B V  4MYWA                 37    0.147  22.640  -0.585   0.052   0.084   0.864
E K  4MYWA                 38    0.489 100.484  -0.032   0.064   0.216   0.721
E R  4MYWA                 39    0.331  75.891  -0.099   0.074   0.484   0.442
B V  4MYWA                 40    0.216  33.138  -0.444   0.023   0.655   0.322
B Y  4MYWA                 41    0.112  23.870   0.304   0.023   0.655   0.322
B H  4MYWA                 42    0.288  52.333  -0.233   0.022   0.552   0.426
B I  4MYWA                 43    0.246  45.473  -1.029   0.021   0.451   0.528
E Q  4MYWA                 44    0.476  84.924  -0.139   0.021   0.279   0.699
E P  4MYWA                 45    0.480  68.055  -0.679   0.019   0.141   0.840
E S  4MYWA                 46    0.527  61.729  -0.514   0.018   0.047   0.935
B L  4MYWA                 47    0.124  22.686   0.184   0.018   0.047   0.935
E E  4MYWA                 48    0.348  60.813  -0.207   0.018   0.019   0.964
E D  4MYWA                 49    0.426  61.430  -0.345   0.018   0.019   0.964
B P  4MYWA                 50    0.233  33.049  -1.144   0.058   0.017   0.925
B F  4MYWA                 51    0.197  39.578  -1.400   0.058   0.017   0.925
E Q  4MYWA                 52    0.494  88.193  -0.573   0.058   0.017   0.925
E P  4MYWA                 53    0.445  63.131  -1.151   0.018   0.019   0.964
E P  4MYWA                 54    0.513  72.795  -1.762   0.018   0.019   0.964
E S  4MYWA                 55    0.740  86.705  -1.491   0.018   0.047   0.935
E I  4MYWA                 56    0.377  69.800   0.211   0.020   0.205   0.775
E P  4MYWA                 57    0.429  60.875  -0.647   0.005   0.262   0.733
B I  4MYWA                 58    0.118  21.775   0.032   0.004   0.514   0.481
E T  4MYWA                 59    0.414  57.380   0.221   0.004   0.616   0.381
B V  4MYWA                 60    0.113  17.337   1.154   0.021   0.756   0.223
B Y  4MYWA                 61    0.219  46.843   0.729   0.018   0.846   0.136
B Y  4MYWA                 62    0.101  21.520   0.792   0.018   0.846   0.136
B A  4MYWA                 63    0.046   5.113  -0.087   0.011   0.918   0.071
B V  4MYWA                 64    0.123  18.905   0.297   0.018   0.846   0.136
B L  4MYWA                 65    0.117  21.423  -0.517   0.021   0.756   0.223
E E  4MYWA                 66    0.285  49.702  -0.718   0.022   0.359   0.619
E R  4MYWA                 67    0.364  83.333  -0.248   0.018   0.088   0.893
E A  4MYWA                 68    0.304  33.512  -0.427   0.018   0.088   0.893
B C  4MYWA                 69    0.029   4.029   0.028   0.004   0.197   0.799
B R  4MYWA                 70    0.172  39.502  -0.609   0.004   0.514   0.481
B S  4MYWA                 71    0.082   9.575   0.323   0.018   0.846   0.136
B V  4MYWA                 72    0.031   4.795   0.211   0.011   0.918   0.071
B L  4MYWA                 73    0.028   5.200   0.181   0.011   0.918   0.071
B L  4MYWA                 74    0.032   5.841  -0.397   0.018   0.846   0.136
B H  4MYWA                 75    0.102  18.554   0.292   0.004   0.514   0.481
B A  4MYWA                 76    0.109  12.001  -0.171   0.005   0.262   0.733
E P  4MYWA                 77    0.253  35.972   0.365   0.018   0.088   0.893
E S  4MYWA                 78    0.330  38.641  -1.773   0.058   0.017   0.925
E E  4MYWA                 79    0.522  91.228  -1.299   0.109   0.005   0.886
B A  4MYWA                 80    0.071   7.879  -1.058   0.600   0.003   0.397
E P  4MYWA                 81    0.301  42.669  -0.871   0.782   0.003   0.216
E Q  4MYWA                 82    0.298  53.205   0.105   0.858   0.002   0.139
B I  4MYWA                 83    0.076  14.041   0.419   0.879   0.010   0.111
B V  4MYWA                 84    0.043   6.532   0.617   0.879   0.010   0.111
B R  4MYWA                 85    0.215  49.166   0.846   0.879   0.010   0.111
B G  4MYWA                 86    0.085   6.674  -0.598   0.938   0.007   0.055
B A  4MYWA                 87    0.041   4.496  -0.209   0.938   0.007   0.055
B S  4MYWA                 88    0.230  26.956   0.317   0.938   0.007   0.055
E D  4MYWA                 89    0.350  50.464  -0.661   0.938   0.007   0.055
E E  4MYWA                 90    0.381  66.543  -0.915   0.858   0.002   0.139
E A  4MYWA                 91    0.352  38.779  -0.898   0.600   0.003   0.397
E R  4MYWA                 92    0.639 146.285  -0.376   0.246   0.004   0.750
E K  4MYWA                 93    0.644 132.368   0.254   0.018   0.019   0.964
E H  4MYWA                 94    0.445  81.000   0.510   0.018   0.088   0.893
E T  4MYWA                 95    0.471  65.258   0.720   0.022   0.359   0.619
B Y  4MYWA                 96    0.072  15.472   0.248   0.004   0.514   0.481
E N  4MYWA                 97    0.336  49.205   0.802   0.004   0.514   0.481
B L  4MYWA                 98    0.035   6.482  -0.058   0.002   0.816   0.182
B T  4MYWA                 99    0.113  15.631   0.340   0.011   0.918   0.071
B I  4MYWA                100    0.028   5.180   0.861   0.006   0.962   0.032
B A  4MYWA                101    0.043   4.728   0.195   0.006   0.962   0.032
B W  4MYWA                102    0.037   8.802  -0.003   0.011   0.918   0.071
B Y  4MYWA                103    0.060  12.843   0.628   0.011   0.918   0.071
B R  4MYWA                104    0.171  39.067   0.830   0.006   0.962   0.032
B M  4MYWA                105    0.093  18.609  -0.057   0.002   0.816   0.182
B G  4MYWA                106    0.223  17.550  -0.611   0.021   0.279   0.699
E D  4MYWA                107    0.694 100.005   0.334   0.005   0.045   0.951
E N  4MYWA                108    0.439  64.270  -0.774   0.005   0.045   0.951
B C  4MYWA                109    0.069   9.617  -0.395   0.021   0.279   0.699
B A  4MYWA                110    0.113  12.409  -0.725   0.023   0.655   0.322
B I  4MYWA                111    0.130  24.050   1.379   0.023   0.655   0.322
B P  4MYWA                112    0.065   9.266   0.266   0.023   0.655   0.322
B I  4MYWA                113    0.030   5.587   0.381   0.021   0.756   0.223
B T  4MYWA                114    0.103  14.300   0.212   0.072   0.855   0.073
B V  4MYWA                115    0.131  20.212   0.853   0.085   0.775   0.140
B M  4MYWA                116    0.112  22.511   0.879   0.157   0.773   0.069
B E  4MYWA                117    0.126  21.995  -0.398   0.172   0.690   0.138
B Y  4MYWA                118    0.122  26.007  -0.098   0.159   0.516   0.325
E T  4MYWA                119    0.266  36.853  -0.423   0.135   0.317   0.548
E E  4MYWA                120    0.558  97.448   0.288   0.064   0.216   0.721
B C  4MYWA                121    0.051   7.118  -0.077   0.018   0.088   0.893
E P  4MYWA                122    0.492  69.815  -0.393   0.018   0.047   0.935
E Y  4MYWA                123    0.419  89.604  -0.357   0.018   0.047   0.935
E N  4MYWA                124    0.643  94.164   0.254   0.053   0.043   0.903
E K  4MYWA                125    0.412  84.728   0.203   0.053   0.043   0.903
E S  4MYWA                126    0.416  48.743  -0.244   0.052   0.084   0.864
B L  4MYWA                127    0.168  30.706  -0.673   0.056   0.142   0.802
E G  4MYWA                128    0.279  21.997  -1.536   0.064   0.216   0.721
E V  4MYWA                129    0.381  58.621   0.175   0.066   0.296   0.638
B C  4MYWA                130    0.035   4.914  -0.256   0.066   0.296   0.638
E P  4MYWA                131    0.386  54.759   0.314   0.069   0.386   0.545
E I  4MYWA                132    0.401  74.167  -0.754   0.021   0.279   0.699
E R  4MYWA                133    0.376  86.081   0.340   0.020   0.205   0.775
B T  4MYWA                134    0.249  34.481  -0.654   0.018   0.047   0.935
E Q  4MYWA                135    0.346  61.849  -0.799   0.058   0.017   0.925
E P  4MYWA                136    0.434  61.556  -1.481   0.113   0.043   0.844
B R  4MYWA                137    0.208  47.609  -0.100   0.191   0.086   0.723
B W  4MYWA                138    0.078  18.687  -0.731   0.216   0.235   0.548
E S  4MYWA                139    0.369  43.282  -0.087   0.216   0.235   0.548
B Y  4MYWA                140    0.171  36.479   0.003   0.231   0.330   0.439
B Y  4MYWA                141    0.090  19.297   0.139   0.216   0.235   0.548
B D  4MYWA                142    0.112  16.197  -0.328   0.216   0.235   0.548
E S  4MYWA                143    0.294  34.480  -1.200   0.125   0.227   0.648
B F  4MYWA                144    0.178  35.684  -0.730   0.125   0.227   0.648
B S  4MYWA                145    0.122  14.287  -1.116   0.066   0.296   0.638
B A  4MYWA                146    0.113  12.508  -0.319   0.066   0.296   0.638
B V  4MYWA                147    0.195  30.018  -0.128   0.066   0.296   0.638
B S  4MYWA                148    0.227  26.640  -0.107   0.019   0.141   0.840
E E  4MYWA                149    0.581 101.536  -0.710   0.053   0.043   0.903
E D  4MYWA                150    0.479  68.966  -0.680   0.053   0.043   0.903
E N  4MYWA                151    0.285  41.783  -0.576   0.052   0.084   0.864
B L  4MYWA                152    0.063  11.535   0.260   0.066   0.296   0.638
B G  4MYWA                153    0.078   6.115   0.239   0.087   0.683   0.230
B F  4MYWA                154    0.037   7.406   0.349   0.018   0.846   0.136
B L  4MYWA                155    0.061  11.206   0.228   0.011   0.918   0.071
B M  4MYWA                156    0.037   7.404   0.013   0.018   0.846   0.136
B H  4MYWA                157    0.045   8.185   0.013   0.021   0.756   0.223
B A  4MYWA                158    0.048   5.323  -0.220   0.021   0.451   0.528
B P  4MYWA                159    0.092  13.012  -0.085   0.019   0.141   0.840
B A  4MYWA                160    0.164  18.018  -0.118   0.018   0.047   0.935
E F  4MYWA                161    0.407  81.585  -0.175   0.113   0.043   0.844
E E  4MYWA                162    0.373  65.111  -0.897   0.113   0.043   0.844
E T  4MYWA                163    0.318  44.107  -1.357   0.053   0.043   0.903
E A  4MYWA                164    0.545  60.026  -0.311   0.052   0.084   0.864
E G  4MYWA                165    0.250  19.691  -1.456   0.020   0.205   0.775
E T  4MYWA                166    0.264  36.561   0.880   0.004   0.616   0.381
B Y  4MYWA                167    0.094  20.045   0.197   0.002   0.816   0.182
B L  4MYWA                168    0.138  25.341   0.666   0.001   0.900   0.099
B R  4MYWA                169    0.080  18.274   0.289   0.001   0.959   0.040
B L  4MYWA                170    0.037   6.830   0.039   0.001   0.959   0.040
B V  4MYWA                171    0.026   4.073   1.069   0.001   0.959   0.040
E K  4MYWA                172    0.274  56.403   0.979   0.000   0.983   0.017
B I  4MYWA                173    0.048   8.843   0.314   0.001   0.900   0.099
E N  4MYWA                174    0.540  79.085  -0.723   0.005   0.045   0.951
E D  4MYWA                175    0.587  84.616  -0.589   0.005   0.015   0.979
E W  4MYWA                176    0.364  87.662  -0.085   0.004   0.616   0.381
B T  4MYWA                177    0.188  26.034   0.325   0.001   0.900   0.099
B E  4MYWA                178    0.244  42.574   0.656   0.001   0.900   0.099
B I  4MYWA                179    0.134  24.864   0.809   0.001   0.959   0.040
B T  4MYWA                180    0.061   8.516   0.147   0.001   0.900   0.099
B Q  4MYWA                181    0.145  25.933   0.113   0.001   0.900   0.099
B F  4MYWA                182    0.044   8.730   0.543   0.001   0.959   0.040
B I  4MYWA                183    0.074  13.709   0.054   0.001   0.959   0.040
B L  4MYWA                184    0.039   7.123   0.218   0.001   0.959   0.040
E E  4MYWA                185    0.274  47.833   0.642   0.001   0.900   0.099
B H  4MYWA                186    0.093  16.899   0.986   0.003   0.718   0.279
E R  4MYWA                187    0.410  93.776   0.039   0.005   0.262   0.733
E A  4MYWA                188    0.504  55.552  -1.440   0.004   0.085   0.910
E R  4MYWA                189    0.647 148.049  -0.193   0.004   0.138   0.858
B A  4MYWA                190    0.246  27.065  -0.718   0.005   0.262   0.733
E S  4MYWA                191    0.486  56.912   0.844   0.021   0.451   0.528
B C  4MYWA                192    0.034   4.774  -0.144   0.021   0.756   0.223
E K  4MYWA                193    0.371  76.356   1.082   0.021   0.756   0.223
B Y  4MYWA                194    0.092  19.554  -0.113   0.021   0.756   0.223
B A  4MYWA                195    0.246  27.087  -2.043   0.021   0.451   0.528
E L  4MYWA                196    0.378  69.267  -0.241   0.021   0.279   0.699
E P  4MYWA                197    0.577  81.905  -0.674   0.064   0.216   0.721
E L  4MYWA                198    0.370  67.802  -0.928   0.019   0.141   0.840
E R  4MYWA                199    0.565 129.362  -1.024   0.019   0.141   0.840
B I  4MYWA                200    0.211  39.127  -0.084   0.019   0.141   0.840
E P  4MYWA                201    0.447  63.500   0.290   0.018   0.047   0.935
B P  4MYWA                202    0.257  36.497  -0.386   0.018   0.047   0.935
E A  4MYWA                203    0.690  76.027   0.171   0.052   0.084   0.864
E A  4MYWA                204    0.248  27.385   0.709   0.052   0.084   0.864
B C  4MYWA                205    0.025   3.552  -1.251   0.056   0.142   0.802
B L  4MYWA                206    0.201  36.876   0.637   0.019   0.141   0.840
E T  4MYWA                207    0.476  66.091   0.680   0.018   0.088   0.893
B S  4MYWA                208    0.222  25.983  -1.140   0.053   0.043   0.903
E K  4MYWA                209    0.652 134.075  -0.017   0.113   0.043   0.844
E A  4MYWA                210    0.352  38.779   0.095   0.113   0.087   0.800
B Y  4MYWA                211    0.086  18.421  -0.239   0.118   0.150   0.732
E Q  4MYWA                212    0.404  72.137   0.330   0.125   0.227   0.648
E Q  4MYWA                213    0.541  96.587   0.544   0.118   0.150   0.732
B G  4MYWA                214    0.266  20.926  -1.287   0.056   0.142   0.802
E V  4MYWA                215    0.339  52.120  -0.538   0.069   0.386   0.545
E T  4MYWA                216    0.317  43.940  -0.511   0.079   0.592   0.329
B V  4MYWA                217    0.168  25.745  -0.428   0.079   0.592   0.329
B D  4MYWA                218    0.279  40.204  -0.331   0.074   0.484   0.442
B S  4MYWA                219    0.117  13.701  -0.905   0.079   0.592   0.329
B I  4MYWA                220    0.121  22.348   0.063   0.079   0.592   0.329
B G  4MYWA                221    0.100   7.878  -1.152   0.079   0.592   0.329
B M  4MYWA                222    0.062  12.366   0.140   0.069   0.386   0.545
B L  4MYWA                223    0.039   7.086   0.270   0.125   0.227   0.648
E P  4MYWA                224    0.354  50.162  -0.457   0.118   0.150   0.732
B R  4MYWA                225    0.292  66.845  -0.948   0.053   0.043   0.903
B F  4MYWA                226    0.275  55.293  -1.090   0.053   0.043   0.903
B I  4MYWA                227    0.205  37.944   0.160   0.058   0.017   0.925
E P  4MYWA                228    0.292  41.449   0.091   0.339   0.016   0.645
E E  4MYWA                229    0.581 101.518   0.825   0.694   0.003   0.303
B N  4MYWA                230    0.179  26.206  -0.530   0.782   0.003   0.216
B Q  4MYWA                231    0.051   9.019  -0.435   0.858   0.002   0.139
E R  4MYWA                232    0.287  65.838   0.730   0.938   0.007   0.055
E T  4MYWA                233    0.322  44.675   1.078   0.938   0.007   0.055
B V  4MYWA                234    0.049   7.470   0.907   0.938   0.007   0.055
B A  4MYWA                235    0.021   2.270   1.383   0.950   0.028   0.022
E L  4MYWA                236    0.267  48.924   0.832   0.950   0.028   0.022
B Y  4MYWA                237    0.169  36.030   0.983   0.950   0.028   0.022
B S  4MYWA                238    0.033   3.844   0.029   0.950   0.028   0.022
B L  4MYWA                239    0.063  11.554   0.694   0.950   0.028   0.022
E K  4MYWA                240    0.313  64.446   0.807   0.911   0.033   0.057
E I  4MYWA                241    0.311  57.572   0.034   0.831   0.044   0.125
B A  4MYWA                242    0.220  24.266  -0.142   0.522   0.016   0.462
E G  4MYWA                243    0.319  25.090  -1.441   0.115   0.016   0.868
E W  4MYWA                244    0.377  90.620  -1.405   0.018   0.019   0.964
E H  4MYWA                245    0.411  74.725  -1.257   0.018   0.047   0.935
B G  4MYWA                246    0.110   8.641  -0.853   0.018   0.088   0.893
E P  4MYWA                247    0.329  46.685  -0.679   0.018   0.088   0.893
E K  4MYWA                248    0.381  78.269  -0.421   0.018   0.088   0.893
B P  4MYWA                249    0.203  28.735   0.377   0.018   0.047   0.935
B P  4MYWA                250    0.260  36.908  -0.710   0.052   0.084   0.864
B Y  4MYWA                251    0.213  45.539  -0.511   0.056   0.142   0.802
E T  4MYWA                252    0.478  66.243  -0.625   0.064   0.216   0.721
B S  4MYWA                253    0.273  31.949  -1.164   0.064   0.216   0.721
E T  4MYWA                254    0.464  64.301  -0.744   0.066   0.296   0.638
E L  4MYWA                255    0.293  53.630  -1.287   0.020   0.205   0.775
B L  4MYWA                256    0.246  45.043  -0.006   0.019   0.141   0.840
E P  4MYWA                257    0.403  57.228  -0.781   0.018   0.047   0.935
E P  4MYWA                258    0.325  46.160  -1.290   0.053   0.043   0.903
E E  4MYWA                259    0.480  83.839  -0.202   0.113   0.087   0.800
B L  4MYWA                260    0.201  36.766  -0.980   0.191   0.086   0.723
E S  4MYWA                261    0.375  43.997  -0.760   0.278   0.093   0.628
E D  4MYWA                262    0.481  69.327  -1.266   0.354   0.048   0.598
E T  4MYWA                263    0.301  41.818  -1.555   0.268   0.043   0.689
E T  4MYWA                264    0.580  80.446  -2.240   0.181   0.016   0.803
E N  4MYWA                265    0.641  93.901  -1.278   0.058   0.017   0.925
E A  4MYWA                266    0.271  29.875   0.255   0.058   0.017   0.925
E T  4MYWA                267    0.606  84.108  -1.379   0.018   0.019   0.964
E Q  4MYWA                268    0.489  87.246  -0.212   0.018   0.019   0.964
B P  4MYWA                269    0.230  32.594  -1.379   0.018   0.019   0.964
E E  4MYWA                270    0.674 117.678  -0.333   0.018   0.019   0.964
E L  4MYWA                271    0.383  70.109  -1.974   0.018   0.019   0.964
E V  4MYWA                272    0.381  58.498  -0.311   0.018   0.019   0.964
E P  4MYWA                273    0.668  94.732  -0.856   0.058   0.017   0.925
E E  4MYWA                274    0.730 127.461  -0.644   0.058   0.017   0.925
E D  4MYWA                275    0.527  75.955  -1.063   0.058   0.017   0.925
E P  4MYWA                276    0.475  67.374  -0.753   0.115   0.016   0.868
E E  4MYWA                277    0.597 104.331   0.470   0.115   0.016   0.868
E D  4MYWA                278    0.565  81.474  -1.052   0.115   0.016   0.868
E S  4MYWA                279    0.643  75.371  -2.126   0.115   0.016   0.868
E A  4MYWA                280    0.527  58.108  -0.365   0.113   0.043   0.844
E L  4MYWA                281    0.427  78.147  -0.286   0.113   0.043   0.844
B L  4MYWA                282    0.240  43.871  -1.171   0.113   0.087   0.800
E E  4MYWA                283    0.659 115.180  -0.664   0.113   0.087   0.800
B D  4MYWA                284    0.294  42.337  -1.066   0.053   0.043   0.903
E P  4MYWA                285    0.378  53.596  -2.418   0.053   0.043   0.903
E A  4MYWA                286    0.520  57.271  -1.121   0.052   0.084   0.864
B G  4MYWA                287    0.260  20.478  -1.630   0.056   0.142   0.802
E T  4MYWA                288    0.516  71.514  -0.053   0.056   0.142   0.802
E H  4MYWA                289    0.332  60.427  -0.838   0.056   0.142   0.802
E H  4MYWA                290    0.460  83.601  -0.810   0.056   0.142   0.802
E H  4MYWA                291    0.450  81.928  -1.077   0.056   0.142   0.802
E H  4MYWA                292    0.462  84.020  -1.342   0.056   0.142   0.802
E H  4MYWA                293    0.517  93.988  -1.449   0.052   0.084   0.864
E H  4MYWA                294    0.825 150.067  -1.230   0.003   0.003   0.994

Explain the output.  Go back.
```

---
